# Supplementary material for: Relationships between Psychoeducational Rehabilitation and Health Outcomes—A Systematic Review Focused on Acute Coronary Syndrome
Source: J Pers Med. 2021 May 21;11(6):440. doi: 10.3390/jpm11060440 (PMC8223782; doi:10.3390/jpm11060440)
Supplement: Supplementary file 1 [file jpm-11-00440-s001.zip › jpm-1214351-SI.pdf]

Table S1. PRISMA 2009 Checklist

Section/topic	#	Checklist item	Reported on page #
**TITLE**			
Title	1	Identify the report as a systematic review, meta-analysis, or both.	1
**ABSTRACT**			
Structured summary	2	Provide a structured summary including, as applicable: background; objectives; data sources; study eligibility criteria, participants, and interventions; study appraisal and synthesis methods; results; limitations; conclusions and implications of key findings; systematic review registration number.	1
**INTRODUCTION**			
Rationale	3	Describe the rationale for the review in the context of what is already known.	2
Objectives	4	Provide an explicit statement of questions being addressed with reference to participants, interventions, comparisons, outcomes, and study design (PICOS).	2,3
**METHODS**			
Protocol and registration	5	Indicate if a review protocol exists, if and where it can be accessed (e.g., Web address), and, if available, provide registration information including registration number.	3
Eligibility criteria	6	Specify study characteristics (e.g., PICOS, length of follow-up) and report characteristics (e.g., years considered, language, publication status) used as criteria for eligibility, giving rationale.	6, 7
Information sources	7	Describe all information sources (e.g., databases with dates of coverage, contact with study authors to identify additional studies) in the search and date last searched.	3
Search	8	Present full electronic search strategy for at least one database, including any limits used, such that it could be repeated.	3
Study selection	9	State the process for selecting studies (i.e., screening, eligibility, included in systematic review, and, if applicable, included in the meta-analysis).	4
Data collection process	10	Describe method of data extraction from reports (e.g., piloted forms, independently, in duplicate) and any processes for obtaining and confirming data from investigators.	5
Data items	11	List and define all variables for which data were sought (e.g., PICOS, funding sources) and any assumptions and simplifications made.	6, 7
Risk of bias in individual studies	12	Describe methods used for assessing risk of bias of individual studies (including specification of whether this was done at the study or outcome level), and how this information is to be used in any data synthesis.	5, SM (Table S2)
Summary measures	13	State the principal summary measures (e.g., risk ratio, difference in means).	5
Synthesis of results	14	Describe the methods of handling data and combining results of studies, if done, including measures of consistency (e.g., I^2) for each meta-analysis.	Not applicable

Section/topic	#	Checklist item	Reported on page #
Risk of bias across studies	15	Specify any assessment of risk of bias that may affect the cumulative evidence (e.g., publication bias, selective reporting within studies).	5, SM (Figure S1)
Additional analyses	16	Describe methods of additional analyses (e.g., sensitivity or subgroup analyses, meta-regression), if done, indicating which were pre-specified.	Not applicable
**RESULTS**			
Study selection	17	Give numbers of studies screened, assessed for eligibility, and included in the review, with reasons for exclusions at each stage, ideally with a flow diagram.	5
Study characteristics	18	For each study, present characteristics for which data were extracted (e.g., study size, PICOS, follow-up period) and provide the citations.	6, 7
Risk of bias within studies	19	Present data on risk of bias of each study and, if available, any outcome level assessment (see item 12).	SM (Table S2)
Results of individual studies	20	For all outcomes considered (benefits or harms), present, for each study: (a) simple summary data for each intervention group (b) effect estimates and confidence intervals, ideally with a forest plot.	8 – 14, SM (Table S3)
Synthesis of results	21	Present results of each meta-analysis done, including confidence intervals and measures of consistency.	Not applicable
Risk of bias across studies	22	Present results of any assessment of risk of bias across studies (see Item 15).	SM (Figure S1)
Additional analysis	23	Give results of additional analyses, if done (e.g., sensitivity or subgroup analyses, meta-regression [see Item 16]).	Not applicable
**DISCUSSION**			
Summary of evidence	24	Summarize the main findings including the strength of evidence for each main outcome; consider their relevance to key groups (e.g., healthcare providers, users, and policy makers).	14, 15
Limitations	25	Discuss limitations at study and outcome level (e.g., risk of bias), and at review-level (e.g., incomplete retrieval of identified research, reporting bias).	15
Conclusions	26	Provide a general interpretation of the results in the context of other evidence, and implications for future research.	16
**FUNDING**			
Funding	27	Describe sources of funding for the systematic review and other support (e.g., supply of data); role of funders for the systematic review.	16

Table S2. Methodological quality summary: review authors' judgements about each methodological quality item for each included study

	Random sequence generation (selection bias)	Allocation concealment (selection bias)	Selective reporting (reporting bias)	Were groups balanced at baseline? (other source of bias)	Intention to treat analysis (other source of bias)	Did both groups receive comparable care (other source of bias)	Blinding -participants and personnel (performance bias)	Blinding outcome assessment (detection bias)	Incomplete outcome data (attrition bias)
Chiavarino 2017	?	+	+	−	+	−	+	+	+
Davidson 2010	+	+	+	+	+	+	−	−	+
Fernandes 2017	+	?	+	−	+	−	+	+	−
Huffman 2019	+	−	+	+	+	+	+	?	+
Nasiri 2020	+	−	+	+	+	−	+	+	?
Norlund 2018	+	+	+	+	−	+	+	−	+
O'Brien 2014	+	+	+	+	−	?	+	+	+
O'Neil 2015	+	+	+	+	−	+	+	−	+
Oranta et al. 2010	+	?	+	+	+	−	+	+	?
Roncella 2013	?	+	+	+	−	+	+	+	+
Sunamura 2017	+	+	−	+	?	+	+	+	+

Figure S1. Risk of bias across studies, N = 11

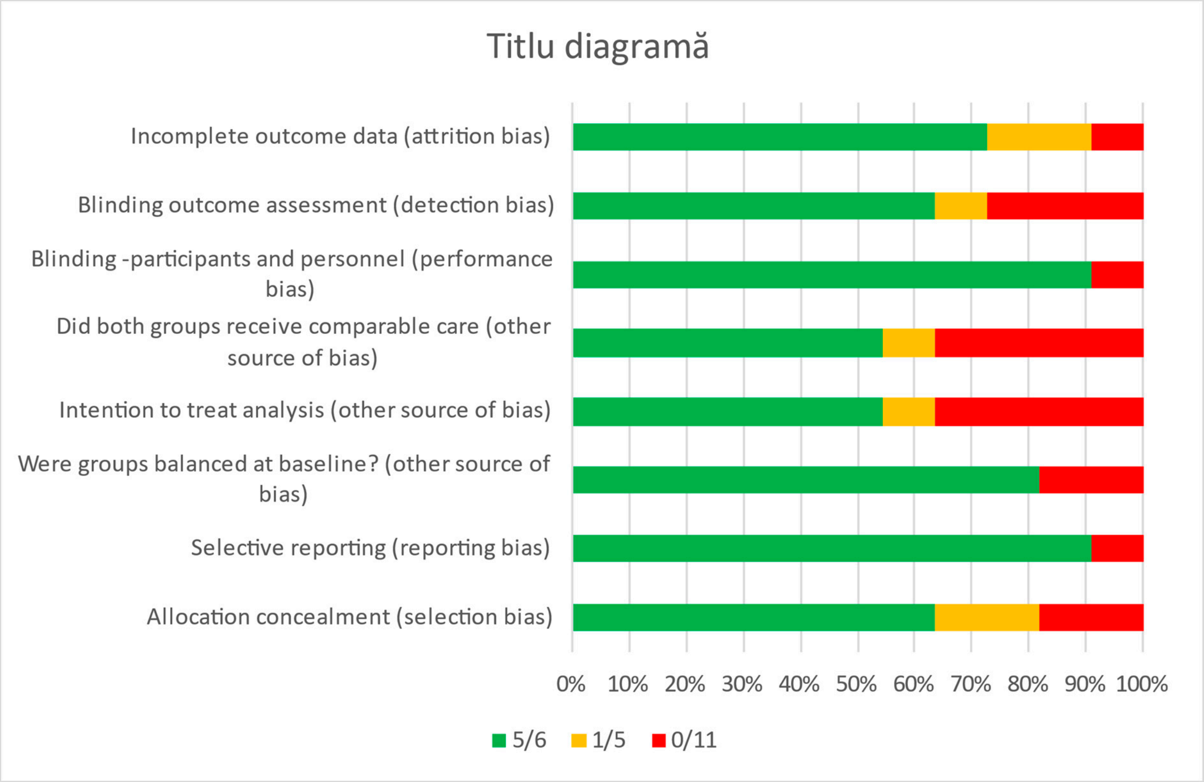

Table S3. Outcomes summary of outcomes for each included study

Outcome	Measure	P value	Effect size at maxim follow-up
**CHIVARINO et al. 2016**			
Quality of life	World Health Organization Quality of Life Brief:		
	total score	**p < 0.001**	F(1, 52): 14.8
	physical health	**p < 0.001**	F(1, 52): 13.3
	psychological health	**p < 0.001**	F(1, 52): 12.3
	social relationships	**p < 0.001**	F(1, 52): 13.7
	environment	**p = 0.026**	F(1, 52): 5.2
Medical variable	Systolic blood pressure	**p = 0.019**	F(1, 58): 5.8
	Diastolic blood pressure	p > 0.265	All F < 1.3
	Low-density lipoprotein cholesterol	**p < 0.001**	F(1, 43): 18.2
	High-density lipoprotein cholesterol	**p < 0.001**	F(1, 43): 28.5
	Triglycerides	**p = 0.047**	F(1, 44): 4.2
	Glycemia	p > 0.123	All F < 2.5
	Creatinine	**p = 0.002**	F(1, 46): 10.3
	Body mass index	p > 0.164	All F < 2.0
	Heart rate	**p = 0.023**	F(1, 56): 5.5
	Ventricular ejection fraction	**p = 0.021**	F(1, 57): 5.7

Psychological variables			
Coping strategies	Brief Coping Orientations to Problems Experienced:		
	total score	**$p = 0.027$**	$F(1, 59)$: 5.2
	emotion-focused subscale	**$p = 0.001$**	$F(1, 59)$: 12.3
	problem-focused subscale	**$p = 0.002$**	$F(1, 59)$: 10.2
	dysfunctional coping subscale	$p > 0.759$	All $F < 0.1$
Emotional awareness	Twenty-Item Toronto Alexithymia Scale:		
	total score	**$p = 0.035$**	$F(1, 58)$: 4.6
	externally oriented thinking subscale	**$p = 0.003$**	$F(1, 58)$: 9.3
	difficulty identifying feelings subsclale	$p > 0.166$	all $F < 2.0$
Self-esteem	difficulty describing feelings	$p > 0.166$	all $F < 2.0$
Health locus of	subscale	$p > 0.652$	all $F < 0.2$
control	General Self-Efficacy Scale	**$p = 0.002$**	$F(1, 29)$: 11.7
	Multidimensional Health Locus of Control Scale		
Behavioral variables	Smoking stopp	$p = 0.361$	NS
	Physical exercise continued	**$p = 0.001$**	
**DAVIDSON et a. 2010**			
Patients satisfaction about the intervention	Patients reporting depression care as excellent or very good	$p < 0.001$	OR (95% CI): 5.4 (2.2- 12.9)
MACE events		**$p = 0.047$**	NS
Depression	Beck Depression Inventory	**$p = 0.005$**	OR (95% CI): 0.59 (0.18- 1.00)
**FERNANDES et al. 2017**			
Emotional state	Portuguese versions of the Hospital Anxiety and Depression Scale:		
	total score	**$p < 0.001$**	M(SD): 5.520 (0.635) vs 28.092 (0.690)
	anxiety	**$p < 0.001$**	M(SD): 2.847 (0.339) vs 12.017 (0.367)
	depression	**$p < 0.001$**	M(SD): 2.623 (0.374 vs 16.134 (0.406)
Illness cognitions	Portuguese versions of the Brief Illness Perception Questionnaire:		M(SD):
	Consequences	**$p < 0.001$**	1.892 (0.060) vs 3.643 (0.065)
	Timeline	**$p < 0.001$**	1.937 (0.070) vs 3.429 (0.075)
	Personal control	**$p < 0.001$**	2.860 (0.094) vs 0.617 (0.102)
	Treatment control	**$p < 0.001$**	3.148 (0.107) vs 2.221 (0.116)
	Identity	**$p < 0.001$**	0.399 (0.092) vs 0.912 (0.100)
	Concern	**$p < 0.001$**	1.757 (0.085) vs 3.550 (0.091)
	Comprehensibility	**$p < 0.001$**	3.394 (0.100) vs 0.728 (0.107)
	Emotions	**$p < 0.001$**	1.493 (0.093) vs 3.499 (0.101)
**FERNANDES et al. 2018**			

Knowledge about ACS	Portuguese versions of the Knowledge Questionnaire	**p = 0.000**	M(SD): 15.70 (0.342) vs 2.92 (0.370)
**NASIRI et al. 2010**			
Perceived stress	Perceived Stress Scale-14	**p < 0.001**	M(SD) = 23.03 (7.79) vs 33.75 (3.78)
Brief illness perception	Brief Illness Perception Questionnaire	**p < 0.001**	
**NORLUND at al. 2018**			
Depression/anxiety	Hospital Anxiety and Depression scale:		
	total score	p = 0.53	ES (95% CI): −0.47 (−1.95 to 1.00)
	anxiety subscale	p = 0.82	ES (95% CI): −0.09 (−0.91 to 0.72)
	depression subscale	p = 0.32	ES (95% CI): −0.45 (−1.34 to 0.44)
	Montgomery-Åsberg Depression Rating Scale-Self rating	p = 0.48	ES (95% CI): −0.58 (−2.20 to 1.04)
	The Behavioral Activation for Depression Scale-Short Form	p = 0.58	ES (95% CI): −0.50 (−2.31 to 1.30)
Cardiac Anxiety	Cardiac Anxiety Questionnaire	p = 0.50	ES (95% CI): −0.73 (−2.83 to 1.38)
**O'BRIEN et al. 2014**			
Knowledge about ACS symptoms	Dichotomous scale (26 items):		
	5 items assessed knowledge of ACS facts (true/false)	**p < 0.001**	NS
	21 items measured recognition of ACS symptoms (yes/no)		F (2,1111): 12.750
Atitude	Attitude scale (5 items)	**p = 0.003**	F (2,1111): 5.111
Belief	Beliefs scale (9 items)	**p < 0.001**	M(SD)(95%CI): 2.22 (4.30)(-2.57 -1.87) vs -1.32 (3.77)(-1.32 -1.64)
**O'NEIL et al. 2015**			
Depression	Cardiac Depression Scale	p = 0.558	ES (95% CI): 3.06 (-7.8 to 4.3)
	Patient Health Questionnaire 9	**p = 0.025**	ES (95% CI): 0.81 (-0.2 to -3.4)
Quality of life	SF-12 Physical component score	p = 0.117	ES (95% CI): 1.42 (-0.6 to 5.1)
	SF-12 Mental component score	p = 0.070	ES (95% CI): 1.74 (-0.3 to 6.6)
**SUNAMURA et al. 2017**			
The 10-year CVD mortality risk	SCORE Risk Score	p = 0.48	ES: 3.30% (25%–75% IQR, 1.01–5.59) vs 3.47% (25%–75% IQR, 0.86– 6.28)
Quality of life	MacNew Questionnaire:		
	Emotional scale	**p = 0.004**	NS
	Physical scale	**p = 0.015**	

Anxiety	Anxiety Score	**p = 0.036**	NS
Cardiovascular risk factors	Smoking<br>Total cholesterol (mmol/L)<br>Systolic blood presure (mmHg)<br>Waist circumference (cm)	**p < 0.001**<br>**p < 0.001**<br>p > 0.05<br>p > 0.05	ES: 13.4% vs 21.3%<br>ES: 3.9 vs 4.3 mmol/L
**TER HOEVE et al. 2018**			
Physical behavior	Step count (nr of steps per min of wear time)<br>Time in prolonged moderate-to-vigorous physical activity	**p = 0.035**<br>**p = 0.002**	ES (95% CI): 0.45 (0.03 to 0.86)<br>ES (95% CI): 1.77 (1.20 to 2.60)
**ORANTA et al. 2010**			
Depression	Beck Depression Inventory	**p = 0.009**	OR (95% CI): 0.31 (0.16 to 0.61) vs 1.15 (0.60 to 0.22)
Distress	Symptom Checklist-25	p = 0.299	OR (95% CI): 0.42 (0.21–0.84) vs 0.90 (0.43–1.86)
**ORANTA et al. 2011**			
Quality of life	EuroQol-5D questionnaires<br>Mobility<br>Self-Care<br>Anxiety/Depression	 p = 0.33<br>p = 0.77<br>p = 0.99	 NS
**ORANTA 2012**			
Use of Healthcare Services	Any specialized healthcare service	**p = 0.007**	NS
**HUFFMAN et al. 2019**			
Physical behavior	Moderate to vigorous physical activity (Accelerometer)<br>Number of steps	**p = 0.026**<br>p = 0.14	EMD: 15.08; ES: 6.75<br>EMD: 1617; ES: 1081
Positive affect	Positive and Negative Affect Schedule	**p < 0.001**	EMD: 7.34; SE: 2.16
Quality of life	SF-12 Physical component score<br>SF-12 Mental component score	p = 0.55<br>p = 0.80	EMD: 4.29; SE: 2.24<br>EMD: 4,44; SE: 2.54
Health behavior	Self-reported health behavior adherence (Medical Outcomes StudySpecific Adherence Scale)	p = 0.22	EMD: 8.91; SE: 3.58
**RONCELLA et al. 2013**			
Combined incidence of new cardiovascular events		**p = 0.0006**	I (%) (N): 21/49 (43%) (33) vs 35/45 (78%) (78)

Cardiovascular events	Reinfarction<br>Death<br>Stroke<br>Revascularization<br>Major adverse cardiac and cerebrovascular events<br>Life-threatening ventricular arrhythmia<br>Recurrence of typical angina	p = 0.67<br><br><br>p = 0.33<br><br>p = 0.24<br><br>p = 0.23<br>**p = 0.04**	I (%) (N) for recurrence of typical angina: 14/49 (29%) (14) vs 22/45 (49%) (23)
New non-cardiovascular events		**p < 0. 0001**	I (%) (N): 5/49 (10%) (5) vs 25/45 (56%) (37)
Re-hospitalizations	Total	**p = 0.02**	M(95% CI): 0.77 (0.53–0.98) vs 1.2 (0.92–1.57)
	Cardiovascular	P = 0.14	M(95% CI): 0.69 (0.48–0.90) vs 1.0 (0.71–1.38)
	Non-Cardiovascular	P = 0.25	M(95% CI): 0.08 (0.002–0.16) vs 0.2 (0.05–0.35)
NYHA class	NYHA class ≥2	**p = 0.01**	I (%): 1/49 (2%) vs 8/45 (18%)
Distress	Self-evaluation test<br>Assessing global psychological distress	p = 0.85	ES(IQR): 5 (3 to 8) vs 5 (3 to 7)
Vital exhaustion	Maastricht Questionnaire	p= 0.32	M(SD): 56.5 (8.1) vs 59. 7 (14.5)
Depression	Beck Depression Inventory	**p = 0.03**	ES(IQR): 6 (3 to 8) vs 8 (5 to 14)
Quality of life	MacNew Questionnaire: Global Score	p = 0.07	ES(IQR): 6.07 (5.48 to 6.39) vs 5.67 (4.89 to 6. 31)
	MacNew Questionnaire: Emotional Score	p = 0.38	ES(IQR): 5.79 (5.36 to 6.35) vs 5.79 (5.0 to 6.32)
	MacNew Questionnaire: Physical Score	**p = 0.03**	ES(IQR): 6.23 (5.70 to 6.53) vs 5.69 (4.85 to 6. 29)
	MacNew Questionnaire: Social Score	p = 0.06	ES(IQR): 6.15 (5.69 to 6.61) vs 5.86 (5.0 to 6.46)
**PRISTIPINO et al. 2019**			
Combined incidence of new cardiovascular events		p = 0.929	I (%) (N): 13/40 (33%) (21) vs 13/36 (36%) (22)

Cardiovascular events	Reinfarction<br>Death<br>Stroke<br>Revascularization<br>Major adverse cardiac and cerebrovascular events<br>Life-threatening ventricular arrhythmia<br>Recurrence of typical angina	p = 1.00<br>p = 1.00<br>-<br>p = 1.00<br>p = 1.00<br><br>p = 1.00<br>p = 0.20	I (%) (N) for recurrence of typical angina: 2/40 (5%) (2) vs 6/36 (17%) (6)
New non-cardiovascular events		p = 1.00	I (%) (N): 11/40 (28%) (15) vs 9/36 (25%) (15)
Re-hospitalizations	Total<br>Cardiovascular<br>Non-Cardiovascular	p = 0.968<br>p = 1.00<br>p = 1.00	Events x 100 patient-year (95% CI) for total re-hospitalizations: 48.0 (38.4 to 57.6) vs 57.4 (45.3 to 69.5)
NYHA class	NYHA class ≥ 2	**p = 0.018**	I (%): 0/43 (4%) vs 6/36 (17%)

MACE = major adverse cardiovascular event; ACS = acute coronary syndrome; CVD = cardiovascular disease; SF-12 = 12-item short form survey; NS = not specified; OR = odds ratio; CI = confidence interval of the difference; M = Mean; SD = standard derviation; ES = effect size; IQR = Interquartile range; EMD = estimated mean difference; I = Incident proportion; N = Nr. of events
